# EEG Assessment in Patients With Disorders of Consciousness: Aims, Advantages, Limits, and Pitfalls

**DOI:** 10.3389/fneur.2021.649849

**Published:** 2021-04-01

**Authors:** Davide Rossi Sebastiano, Giulia Varotto, Davide Sattin, Silvana Franceschetti

**Affiliations:** ^1^Department of Neurophysiopathology, Fondazione I.R.C.C.S. Istituto Neurologico Carlo Besta, Milan, Italy; ^2^Epilepsy Unit, Bioengineering Group, Fondazione I.R.C.C.S. istituto Neurologico Carlo Besta, Milan, Italy; ^3^Department of Neurology, Public Health and Disability, Fondazione I.R.C.C.S. Istituto Neurologico Carlo Besta, Milan, Italy

**Keywords:** disorder of consciousness, quantitative EEG analysis, EEG-connectivity, sleep, coma—rehabilitation

## Abstract

This study presents a brief review of literature exploring simple EEG-polygraphic examinations and procedures that can be carried out at a patient's bedside. These include EEG with a common electrode array and sleep evaluation. The review briefly discusses more complex analytical techniques, such as the application of advanced EEG signal processing methods developed by our research group, to define what type of consistent markers are suitable for clinical use or to better understand complex patient conditions. These advanced analytical techniques aim to detect relevant EEG-based markers that could be useful in evaluating patients and predicting outcomes. These data could contribute to future developments in research.

## Introduction

Analysis of spontaneous EEG activity is an important technique in exploring and evaluating patients with disorders of consciousness (DOCs) in both acute and chronic conditions. Studies and literature on this subject outline the importance of this technique in assessing the severity of brain damage and trying to predict possible patient outcomes (1, 2). In addition to conventional visual inspection of EEG traces, in recent decades a number of analytical algorithms have been applied to resting EEG signals. These algorithms extract quantitative parameters for detecting qualitative changes in the cortico-cortical or cortico-thalamic coupling, as discussed in recent reviews by Bai et al. (3).

The present review provides a brief overview of literature in the field, reporting relevant evidence and discussing new emerging results based on the experiences of our research group.

## “Standard” EEG Recording and Direct “Inspective” Judgment

The relevance of EEG recording in the evaluation of disorders of consciousness has been used since the milestone work of Plum and Posner (4). The EEG is the most common basic instrumental examination for the diagnosis of brain death. It continues to be the only procedure that enables the bedside monitoring of both “immediate” and long-lasting cortical functioning related to the conscious/unconscious “state.” Compared with all other neuroimaging techniques, EEG recordings are a more widely applicable, less expensive, and suitable procedure that provides direct and immediate information. However, limits and misinterpretations can happen due to severe cranial defects caused by previous injuries or operations, the presence of various types of artifacts, and the ICU environment. To date, few studies have investigated the relationship between EEG and cranial defects in DOC, and some research has described the EEG related effects of skull defects in other clinical contexts (5, 6). These factors alongside patient history need to be taken into account in the processing and interpretation of the signal.

The evaluation of “routine” EEG is conducted according to appropriate scales in terms of continuity, voltage amplitude, frequency, and reorganization of an anterior/posterior gradient of the background activity, symmetry, presence of spontaneous variability or reactivity, and presence of epileptic discharges (7). In practice, it is straightforward to distinguish the good organization of a well-modulated EEG signal from a condition in which it appears monotonous, poorly modulated in space, or even presents with well know patterns of severe brain damage (e.g., suppression burst, or minimal or no activity). However, it is not easy to interpret and classify intermediate patterns.

Other studies have examined how to simplify EEG evaluation in DOC patients by proposing a semi-quantitative scale. These include Synek scales (8) and a reorganization proposed by Young et al. (9). These represent the first attempts to standardize EEG evaluation in this field; however, they are subject to limitations in the definition of intermediate patterns. Although both the American Clinical Neurophysiology Society (7) and, more recently, the European Academy of Neurology (10) have proposed guidelines and recommendations that have been successfully applied to long-lasting EEG recordings in selected populations (11), an extensive application of this rule in different clinical contexts is still lacking. This limits useful applications in clinical settings and reduces the homogeneity of the classification criteria necessary for scientific production.

**Figure 1:**
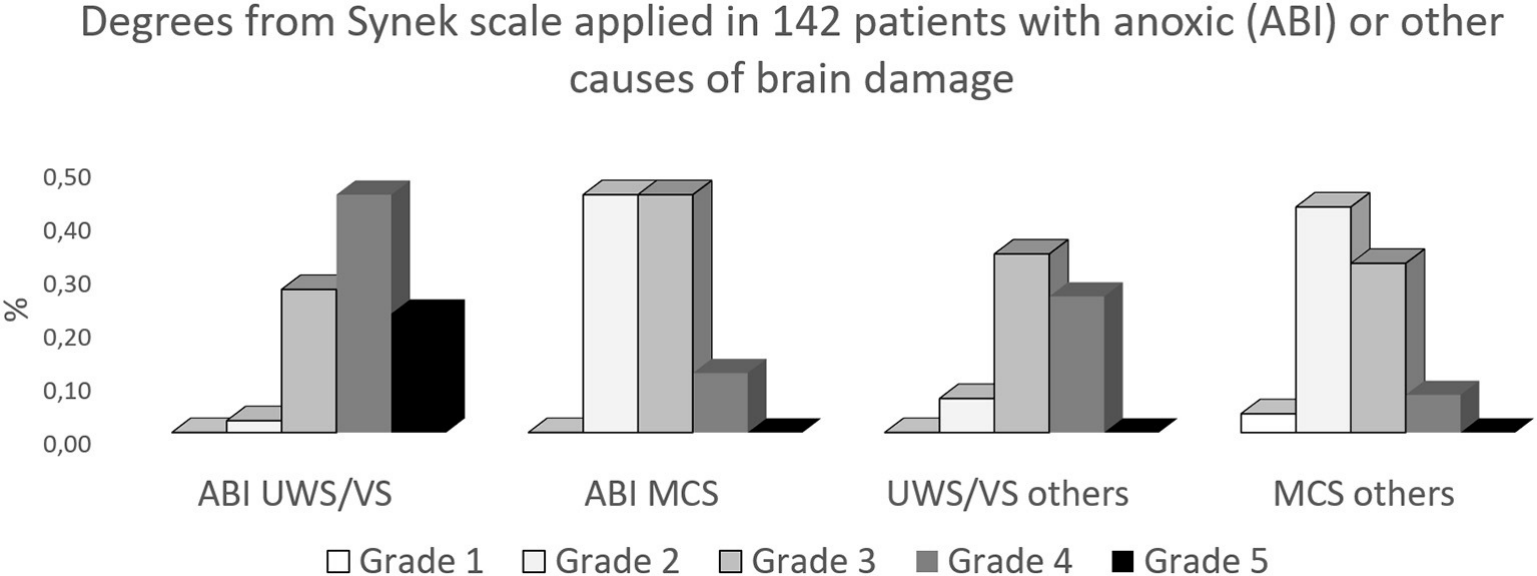
Results from the attempt to apply the Synek scale (8) on resting EEG, obtained from 142 patients with chronic DOC. ABI, anoxic brain damage; others, traumatic or vascular brain damage; (UWS/VS), unresponsive wakefulness syndrome/vegetative state; MCS, minimally conscious state.

Figure 1 describes the results we obtained by applying the Synek scale in 142 patients with different degrees of DOC, more severe degrees (signal attenuation or suppression burst) are prominent in patients classified as having unresponsive wakefulness syndrome/vegetative state (UWS/VS) with anoxic brain damage. As can be seen from Figure 1, intermediate grades were present in different percentages in all conditions.

## EEG Signal Reactivity

The main point evaluated in the EEG of patients with DOC is “reactivity” in response to different external stimuli. A multicenter study by Estraneo et al. (12) was able to predict patient outcomes using EEG reactivity to eye opening and closing and acoustic stimuli, defined as a reproducible change in frequency or amplitude of the signal in a 3-s epoch. The forecast of the outcome was superior to those obtained using tactile and noxious stimuli and evoked potentials, including event-related potential ERP. Chen et al. (13) reported on the significant prognostic value of post-stimulation EEG reactivity at different frequency bands and changes in connectivity function. These were significantly correlated with the outcome when evaluated after “quantifiable” electrical stimulation obtained using 5 Hz square-wave pulses lasting 2 s over the motor threshold. In the current clinical context, EEG reactivity rather simple stimulus protocols offer an easy and noninvasive measure for exploring peripheral and central sensory ascending pathways, providing incomplete but useful information about the functional sparing of the brainstem, thalamus, and the cerebral cortex (14).

## Advanced EEG Analysis

Both simple and more complex techniques have been used to quantify resting (stimulus independent) EEG or changes induced by various stimuli, as outlined in a review by Jain and Ramakrishnan (15). The different approaches include techniques needing an uneven degree of post-processing and elaboration of the signals, including power spectrum analysis, complexity analysis, and entropy or functional connectivity analysis, which are all applicable through appropriate analytical algorithms.

The evaluation of resting-state activity can well investigate the functional architecture of the brain and resting-state networks (16), even if an extensive clinical application is still poorly developed. The earliest studies aimed to express the signal characteristics in terms of frequencies and amplitude, utilizing a simple mathematical function that extracted the power spectrum, and are currently available within the “on board” software of the various polygraphs applied to record the EEG. These rather simple procedures maintain a suitable role in the clinical practice. The ratios between frequencies above 8 Hz and frequencies below 8 Hz were found to significantly correlate with the values of the revised Coma-Recovery Scale (r-CRS) (17, 18). Similarly, in our experience, we found a direct correlation between the alpha relative power and the clinical scores, and an inverse correlation with delta relative power in patients with brain damage due to various etiologies (19).

Spectral analysis adds to the evaluation with numerical indexes, which can be used in patient populations (13). It could also provide information on individual patients who have repeated EEG recordings during follow-up.

It is less easy to apply complex analytical techniques, as often they need time and require a working team with good cooperation between different professional roles, such as medical doctors, technicians, physicists, engineers, mathematicians, which are not always present in a clinical setting. EEG analyses apply to the signals recorded at a patient's bedside and can be used as an alternative to more troubling techniques, for example, those based on functional imaging, requiring sedation, which are often affected by difficult to recognize artifacts. Due to the fact that they are easy to obtain, they can be applied repeatedly during the early post-damage recovery period.

Several studies have been performed on resting EEG, using non-linear analysis based on indices as complexity and entropy [see (15) for review]. Entropy, which is a measure of regularity, offers some particularly intuitive measures that are potentially suitable in providing easily interpretable results in clinical practice, since high entropy values indicate that a subject has a less regular (monotonous) EEG at rest, thus being closer to an awake state, while lower values have been associated with unconscious states (20). Different algorithm have been applied to calculate entropy, such as Lempel-Ziv complexity, approximate entropy, or cross-entropy, with consistent and promising results both to distinguish between UWS/VS and those in the minimal conscious state (MCS) (21) and to correlate with different r-CRS scores (3, 15, 22).

Functional connectivity has also been employed to assess the level of integration of brain networks in patients with DOC. The connectivity of EEG resting state has been analyzed through several methods such as coherence (23), imaginary coherence (IC) (24), phase lag index (PLI) (25), and directed transfer function (26). Whereas, the different methods can show different performances and applicability, with specific advantages and limitations (27) most of these studies converge in the conclusion that EEG network analysis is a suitable approach for distinguishing UWS/VS from MCS patients (see Jain and Ramakrishnan for a review).

Some studies specifically focused on the comparison of different connectivity measures. Höller et al. (26) found that the best results were achieved with partial coherence, but also directed transfer function and generalized partial directed coherence were capable of distinguishing UWS/VS from MCS patients. Lehembre et al. (24) found that IC and PLI were equally suitable in distinguishing UWS/VS from MCS in low density EEG and improved negative results of coherence, which were more affected by volume conduction problems. Stefan et al. (28), combined different indexes including microstates, entropy, power in alpha and delta frequency bands, and connectivity indexes, and found that the percentage of time spent in alpha microstates (epochs of the semi-stable configuration of the scalp potential in different “nodes” of the network) reached the best in distinguishing UWS/VS from MCS patients. Conversely, the clustering coefficient (a measure of the degree to which nodes in a graph tend to cluster together) obtained on beta coherence had a higher value in predicting the outcome.

In our experience, sometimes data required a non-obvious interpretation. In one study performed using partial directed coherence to assess the functional connectivity in 19 UWS/VS patients (29) we found, as expected, that there was a strong reduction of relative power in the alpha band, but the alpha activity was hyper-connected in the central and posterior cortical regions. This increased connectivity was obvious in patients with anoxic brain damage but also those with severe traumatic or vascular damage. If we consider the alpha activity as a marker of “physiological” neuronal oscillations and as a mechanism of integrative functions (30), our finding can be considered a contradictory finding. However, we attributed this data to a remarkable rearrangement of connectivity in patients with chronic UWS/VS, probably deriving from an early neurodegenerative process, followed by a reshaping of intra-cortical connectivity in “disconnected” cortical areas with respect to subcortical control.

## Long Lasting EEG Recordings Including Sleep

Several studies underlined the importance of the EEG/polygraphic long-lasting recording to assess the severity of impairment in DOCs patients by studying the residual patterns of the circadian cycle and sleep in DOC patients (31). Nevertheless, the evaluation of which characteristics of the sleep pattern are most useful in defining the state of consciousness and offering information on the outcome remains non-homogeneous. The characteristics of different sleep stages that have been indicated as correlated with lower severity of brain damage include the presence of spindle sleep, slow-wave sleep pattern, and the duration of the sleep condition (32–36).

**Figure 2:**
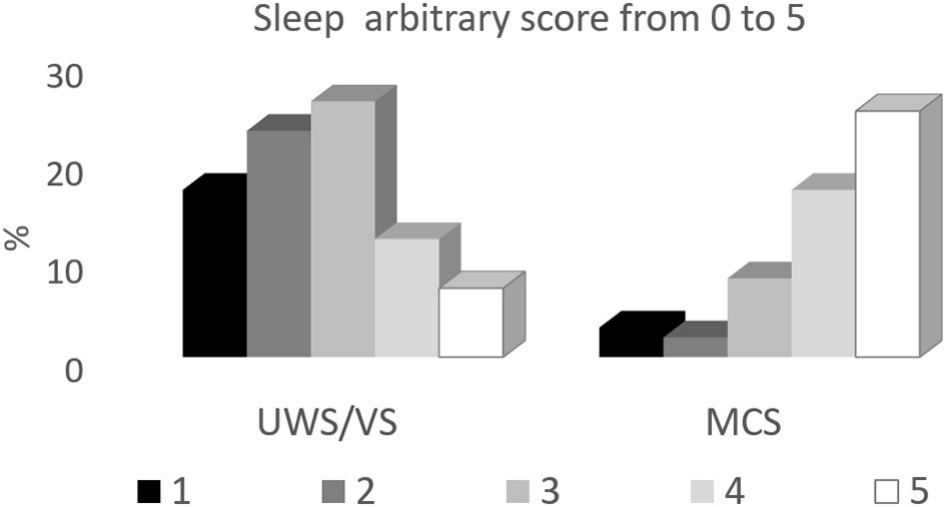
Distribution of the different EEG patterns during behavioral sleep in UWS/VS and MCS patients. The sleep patterns are expressed in percentages with respect to each subgroup, according to the etiology of brain damage (ABI, anoxic brain damage; others, traumatic or vascular brain damage).

Our study included 97 patients in UWS/VS or MCS. The presence of slow wave sleep was higher in percentage in MCS patients both deriving from anoxic and other causes. The presence of sleep circles was especially distinctive, including both spindle and slow wave sleep, while REM pattern could be detected also in more severely damaged patients Figure 2.

Some discrepancies and limitations derive from the heterogeneity of the case series and from the different times at which the patients were evaluated. Different studies evaluated specific features of sleep EEG such as spindles, slow waves, and their circadian regulation, which directly reflect the preservation of fundamental neuronal and network processes, and circuitry oscillations, mainly involving thalamic and cortical circuits (37). In our study, slow-sleep positively correlated with r-CRS and probably, with a more spared cortex, while REM sleep was similarly present in UWS/VS and MCS patients (36). REM occurrence was conversely considered as a positive prognostic factor in subacute conditions (34, 38), while we examined patients in chronic conditions. Moreover, we defined REM sleep based on polygraphic patterns (e.g., appearance of typical rapid eye movements, congruous electrocardiographic, and spirographic parameters), even in the absence of the typical EEG REM patterns. Therefore, in severe conditions, preserved brainstem mechanisms may have sleep patterns similar to that of very immature infants (39).

Moreover, on the topic of EEG evaluation, the literature suggests that, even with some non-uniformity, the preservation of quasi-physiological circadian sleep patterns correlates with better outcomes in the acute/subacute phase, and with higher residual functioning in chronic DOC patients [see (1, 31)].

## Conclusion

Even though there are well-defined “technical languages” and guidelines supporting the application of sufficiently simple methods for qualitative evaluation of standard EEG (7, 10, 40), extensive multicenter studies sharing a common classification method and reaching an agreement are lacking. Despite these limitations, evaluation of EEG recordings remains the simplest method of assessing the severity of brain damage both in acute and chronic stages, even in the presence of some limitations, namely in the evaluation of “intermediate” patterns. Moreover, it allows for the direct evaluation of reactivity to external stimuli. Evoked and event-related potentials can be associated with minimal to moderate extra-time and effort, adding further information that can inform clinical assessment and treatment, as discussed in reviews by Johnson and Kaplan (41), Bai et al. (3), and Comanducci et al. (1).

Long lasting EEG recordings including sleep can probably help in most cases and we strongly support their use, since sleep evaluation and staging provide relevant information about cortical and cortico-subcortical preserved relationships (1, 31). Up to now, there are several automated systems applicable to sleep staging in physiological or mild pathological conditions, but they are often difficult to apply to the disrupted sleep patterns occurring in DOC patients. A suitable consensus on the inspective procedure that homogeneously quantifies and characterizes different sleep stages, based on polygraphic patterns is fundamental in setting-up “targeted” quantitative automatic methods, as proposed by Molteni et al. (42), and needs to be developed and applied more consistently.

Compared to functional “imaging” techniques, which require the patient to move from their bed, which involves a not negligible risk of artifacts in an extraneous environment, especially in patients with less severe impairment of consciousness, “quantitative” EEG analysis techniques have advantages, and often require only easy/basic post-processing. Taking into account that the development of resuscitation techniques in recent years has led to an increase in subjects with severe brain injuries who survive an acute event, the possibility of reaching a consensus on “quantitatively, scoring “circadian” EEG changes would seem fundamental and applicable in “peripheral” hospitals.

This short review included information on the application of EEG at a patient's bedside to increase awareness of the limitations affecting its use, mostly because already existing scores are inconsistently applied or the use of EEG is insufficient. The pitfalls of EEG do not stem from a lack of new methods but from the insufficient homogeneous application of existing methods of evaluating EEG signals both by visual inspection (with a homogeneous style in recording and scoring) and the “advanced” techniques already available, that need to be made more user friendly and validated by a large number analytical techniques.

Although there are excellent reviews, some of which are cited in this text, and many multi-center studies, the importance of DOC in the fields of health and rehabilitation mean that it requires an international consensus. This is needed to be able in regulating the inspective EEG methods, contributing to the “basic” homogeneous evaluation in various centers, both for dedicated centers and for intervention and treatment units present in the territory.

Among various interpretations of the network defects leading to defective consciousness, one of the most explored is the “integrated Information theory” (IITC) (43), which is suitable for predicting markedly reduced states of consciousness, based on neurophysiological and imaging data. The IITC approach, however, represents a very interesting method but is still far from being applied at a patient's bedside.

The many types of post-elaboration procedures show relevant promise for assessments based on quantitative information in clinical settings in the future. Entropy algorithms most likely have the best chance of being applied in individual subjects as well as in population studies, since it is intuitive and potentially user-friendly.

It is more difficult to propose the application of connectivity analysis for extensive use in the near future. This study technique has the advantage of providing precise information on neuronal networks, quantifying damage to them. They are also the most effective method of understanding the extent and type of functional defect but require relevant post-processing. Like other methods of EEG analysis, further extensive studies are required and there needs to be more consistent consensus between different study groups on specific applications depending on patient subsets, including detailed comparisons of different techniques using real as well as simulated signals.

## Author Contributions

SF and DR reviewed the personal data and drafted the paper. GV performed the connectivity analysis and reviewed related literature. DS performed the clinical evaluation of the patients included in the EEG analyses and reviewed the related literature. All authors contributed to the article and approved the submitted version.

## Conflict of Interest

The authors declare that the research was conducted in the absence of any commercial or financial relationships that could be construed as a potential conflict of interest.
